# Classification of pasture habitats by Hungarian herders in a steppe landscape (Hungary)

**DOI:** 10.1186/1746-4269-8-28

**Published:** 2012-08-01

**Authors:** Zsolt Molnár

**Affiliations:** 1 Centre for Ecological Research, Hungarian Academy of Sciences, H-2163, Vácrátót, Alkotmány u. 2-4., Hungary

**Keywords:** Folk biological classification, Phytosociology, Picture sort, Salt steppe, Vegetation continua, Vegetation perception

## Abstract

**Background:**

Landscape ethnoecology focuses on the ecological features of the landscape, how the landscape is perceived, and used by people who live in it. Though studying folk classifications of species has a long history, the comparative study of habitat classifications is just beginning. I studied the habitat classification of herders in a Hungarian steppe, and compared it to classifications of botanists and laymen.

**Methods:**

For a quantitative analysis the picture sort method was used. Twenty-three pictures of 7-11 habitat types were sorted by 25 herders.’Density’ of pictures along the habitat gradient of the Hortobágy salt steppe was set as equal as possible, but pictures differed in their dominant species, wetness, season, etc. Before sorts, herders were asked to describe pictures to assure proper recognition of habitats.

**Results:**

Herders classified the images into three main groups: (1) fertile habitats at the higher parts of the habitat gradient (***partos***, lit. on the shore); (2) saline habitats (***szík***, lit. salt or saline place), and (3) meadows and marshes (***lapos***, lit. flooded) at the lower end of the habitat gradient. Sharpness of delimitation changed along the gradient. Saline habitats were the most isolated from the rest. Botanists identified 6 groups. Laymen grouped habitats in a less coherent way.

As opposed to my expectations, botanical classification was not more structured than that done by herders. I expected and found high correspondence between the classifications by herders, botanists and laymen. All tended to recognize similar main groups: wetlands, ”good grass” and dry/saline habitats. Two main factors could have been responsible for similar classifications: salient features correlated (e.g. salinity recognizable by herders and botanists but not by laymen correlated with the density of grasslands or height of vegetation recognizable also for laymen), or the same salient features were used as a basis for sorting (wetness, and abiotic stress).

**Conclusions:**

Despite all the difficulties of studying habitat classifications (more implicit, more variable knowledge than knowledge on species), conducting landscape ethnoecological research will inevitably reveal a deeper human understanding of biological organization at a supraspecific level, where natural discontinuities are less sharp than at the species or population level.

## Background

Ethnobiologists seek to understand how different peoples perceive, classify, use, and manage the living world [1,2]. In studying the biotic elements of traditional ecological knowledge, ethnobiologists have traditionally focused on the classification and use of plants and animals e.g. [1,3,4]. The comparative study of the landscape ethnoecological semantic domain is just beginning e.g. [5-7]. These studies focus on the perception of landscapes, the parsing of their patterns, and the classification of their constituent parts in local ethnoecological systems [8]. There are two main directions of research. The first focuses on ecological features; namely, how a living landscape is perceived and imagined by local people. It is thus closer to scientific landscape ecology, and is called landscape ethnoecology, [8,9]. The other seeks to understand cultural differences in conceptualisation of landscape by focusing on geography-type physical components in particular such as landforms, water features and vegetation assemblages (ethnophysiography, [10]). Of course, the two directions are mutually connected.

Quantitative analysis of folk habitat knowledge is rare. Sillitoe [11] documented the habitat knowledge of the Wola region of New Guinea and compared it with the scientific vegetation classification. He found, that the two classifications seem to be similar, but the lexical knowledge about these types might be quite different. Fleck and Harder [12] studied the Matses Indians in Peru who distinguish 178 forest types. Vegetation structure, palm species composition, and small mammal fauna of folk habitat types demonstrated the ecological relevance of Matses-recognized habitat types. Abraão et al. [13] prepared a diagram of Baniwa forest classification with corresponding hierarchical ranks sensu [1] of greater or lesser inclusion among classes, and also performed a cluster analysis and a non-metric multidimensional scaling of vegetation types based on informant reports of floristic composition. They emphasized the continuous gradient along which habitats were distinguished. Verlinden and Dayot [14] studied the plant species composition of the habitats distinguished by people in the Kalahari (Namibia). They found, that habitats based on vegetation criteria had little overlap with each other, while habitats with no vegetation criteria often had large overlaps with other habitats. From the temperate regions of Eurasia only one detailed study on folk habitat classification is available [15]. Meilleur reconstructed the highly utilitarian habitat classification of Alpine farmers living in the French Alps. 20 habitats are distinguished based on geographic, topographic, geologic, hydrographic and vegetation criteria.

Landscape elements have a diverse terminology in ethnobiology: ecotope, habitat, kind of place, biotope [16]. I chose to use the term *habitat*, since in Europe this is the most widespread term that includes all living creatures on a piece of land with its soil, bedrock, and hydrology. A habitat is mostly defined by its vegetation, and is more or less a synonym of ecotope (the suggested term by Johnson, Hunn and Meilleur [7,8]).

Habitats in a landscape can be ordered along different gradients [6,17,18]: edaphic gradient (e.g. from wet to dry habitats, from rich soils to poor ones), topographic gradient (e.g. from cold mountain tops to warmer valleys or sea shores), physiognomic gradient (e.g. from grasslands through thickets to forests), successional gradient (habitat patches of different age, e.g. habitats in a slash-and-burn mosaic, or in an abandoned landscape with old-fields and regenerating forests of various age), naturalness gradient (from the most natural to the most anthropogenic ones), and land-use value gradient (from the most used arable fields to hardly used distant forests, or from a fertile pasture to a coarse pasture).

In the studied landscape (Hortobágy steppe, Hungary, Central Europe), the only significant gradient is an edaphic one: distance to groundwater table. Habitats at the higher lying end of this gradient have a deep groundwater table, habitats in the middle have a high water table and are waterlogged for a couple of weeks mostly in spring, while habitats at the lower lying end are usually waterlogged for most of the year. Since there are no forests or rock outcrops, and the landscape is very flat, there are no topographic or physiognomic gradients. Vegetation has been extremely stable for millenia (edaphic paraclimax), and thus there is no successional gradient (except in marginal areas where some abandoned arable fields regenerate in about a decade back to semi-natural steppes). All habitats, such as steppes, meadows, and marshes are dominated by herbaceous species. The land-use value (grass quality) gradient is mostly parallel to the main edaphic gradient. The naturalness gradient is compact, most habitats being semi-natural (most grasslands have never been improved, present species composition is similar to natural). The whole landscape is utilized dominantly as a pasture with some salt meadow hayfields and arable land embedded in the vast steppe. On the other hand, this ”simple” landscape is highly complex. Many habitat patches are transitional in species composition to adjacent patches with sharper or more blurred boundaries. There are usually 3-8 plant communities forming the fine-scale mosaic of the steppe. Moreover, the mosaic is fractal-like: in some cases, individual habitat patches are only several m^2^ in size, while in other places several tens of hectares are covered with one habitat type. The steppe is used by traditional pastoralists. There are ca. 300 active herders in the Hortobágy steppe (an exceptionally high number in Europe) herding mostly sheep and cattle. Ecological knowledge of herders is fairly independent of the scientific botanical or ecological knowledge. Herders have a deep emotional connection to the landscape and the livestock. One often hears repeated: ”I do see richer persons than me, but there is not anybody who is happier”; ”You have to love your animals like you love your family!”.

I argue that this landscape is ideal - from both a cultural and a biological point of view – for the study of the algorithmic details of folk habitat classification. My goal was to obtain a classification of different habitat types that exist in this steppe landscape by picture sort exercises. Sorts are often used to understand people’s perception and structure of a cultural domain and to reveal the underlying perceptual dimensions that people use to distinguish among items [19]. After constructing the habitat classification of herders, I compared it to picture sorts made by botanists and laymen.

Based on previous case studies and theoretical expectations [7,8,20], I had the following expectations. I expected to find a shallow landscape ethnoecological hierarchy in our relatively simple steppe landscape, whereas classification of animal and plant species is usually highly hierarchical [1]. Studies on habitat classification [7,12,21] on the other hand found that habitat classifications are less hierarchical, they are better multidimensional. I expected that the botanists’ classification would be more structured and more hierarchical than the herders’ classification, mostly because in this steppe landscape more than 20 plant associations are described and placed into a multi-layered hierarchy by phytosociologists [22,23]. Classification by laymen was expected to be botanically less explainable, since I believed that many vegetation features recognized by scientists would not be recognized or understood by laymen on the pictures I used. As habitat classifications tend to be ordered into a single multidimensional landscape ethnoecological partition [7], I nevertheless expected substantial correspondence among the three studied classifications.

## Methods

### Study area and its herders

#### The landscape

**Figure 1:**
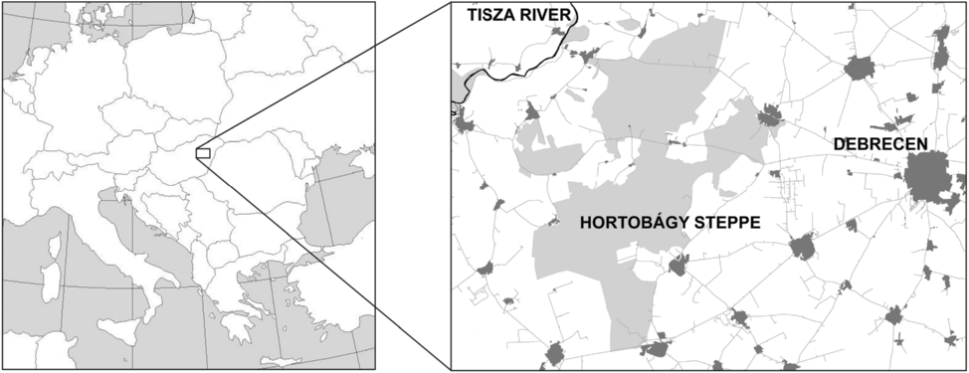
Map of the study area: Hortobágy salt steppe, Hungary.

The Hortobágy steppe (ca. 100 000 hectares) lies in Central Europe, in the Carpathian Basin (Figure 1). The area occurs within the Eurasian forest-steppe belt that spreads from Hungary to Mongolia. In the Pleistocene, the area was a floodplain that gradually dried out and became more and more saline [24]. Water from melting snow and summer rains covers approximately a third of the area for weeks and even months. The entire region is climatically relatively homogeneous, with an average yearly precipitation of appr. 550 mm and a mean annual temperature of appr. 10°C. Snow covers the steppe for appr. 35 days on average in winter. However, the subcontinental climate heavily fluctuates from year-to-year, and consequently water-cover and yearly biomass of the steppe vary highly. The regional flora has ca. 800 vascular plant species including several endemics [22]. The main soil type of the salt steppe is meadow solonetz developed over loess. Groundwater is salty and rich in soda (Na_2_HCO_3_), whereas the groundwater table is relatively high (usually 0.5-2.5 m). The most important dominant and characteristic species of the steppes are *Achillea collina* and *A. setacea, Agrostis stolonifera, Alopecurus pratensis, Artemisia santonicum, Aster tripolium* subsp. *pannonicus, Atriplex tatarica, Beckmannia eruciformis, Bolboschoenus maritimus, Camphorosma annua, Carduus acanthoides, Festuca pseudovina* (and *F. rupicola*), *Hordeum hystrix, Limonium gmelini* subsp. *hungaricum, Matricaria recutita, Phlomis tuberosa, Phragmites australis, Puccinellia limosa, Salvia nemorosa, Schoenoplectus lacustris, Trifolium angulatum* and other annual clover species, *Typha latifolia* and *T. angustifolia.* The vegetation pattern of the steppe is fairly stable: salt steppes have dominated the area since the late Pleistocene [24]. However, river channelizations in the second half of the 19th century decreased regular floods, and drainage works during the 20th century dried out many marshy depressions [23]. The area has been used for extensive grazing for millennia (mostly cattle, horse, later sheep, with some pig and poultry). The area is a refuge for several ancient animal breeds like Hungarian grey cattle, Racka sheep, and Mangalitza pig. Highly saline solonetz soils of the area cannot be improved for arable use. Neither artificial fertilization, nor irrigation or overseeding are profitable in the long run [25]. Herders have had only limited success in improving pastures, e.g. by manuring grasslands at the higher lying end of the edaphic gradient. The main method of pasture improvement used by herders is thorough grazing, which prevents litter accumulation, which would decrease productivity. Even during the socialist regime (1949-1989), when large areas were converted into intensive agriculture in Hungary, many pastures of the salt steppes kept their natural plant species composition, since pasturing techniques were only partly intensified (e.g. by building sheds, motorization of drinking water extraction, modern breeding methods). Today, a National Park established in 1973 preserves traditional ways of herding in the Hortobágy area as a means of nature conservation management. Latin names of plant communities and plants mentioned in the text follows Borhidi and Király [22,26], respectively.

#### The herders

**Figure 2:**
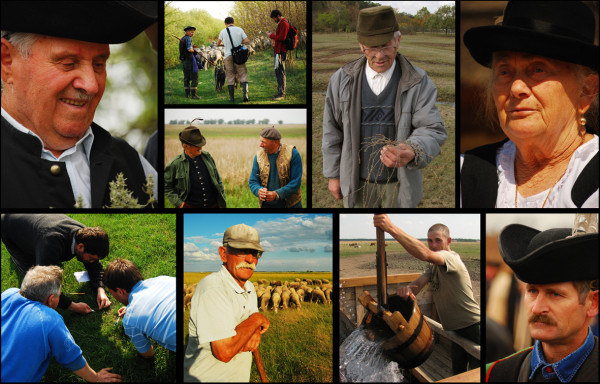
Documentation of a few interviews and interviewees in the Hortobágy steppe.

I present in this paper the habitat classification of Hungarian herders of the Hortobágy salt steppe (Figure 2). Herder communities living in settlements that surround the vast steppe belong to slightly different ethnic groups (kun, hajdú, bihari, debreceni, matyó), and are mostly Calvinists, with some Catholics [27]. Most herders settled in this landscape between the 13^th^ and 18^th^ centuries. Pasturing in this region has its roots in Central Asian nomadic pasturing, since many people arrived from Central Asia to this area before the 13^th^ century (huns, avars, magyars, kuns, jazygs, pechenegs, etc.). Present day similarities among Central Asian and Hungarian herding traditions, however, are mostly the result of similar ecological conditions, namely the steppe environment. Between the 16^th^ and 18^th^ centuries, cattle reared in this area were an important export to Austria and Germany. In the 19^th^ century, sheep herding based mostly on Balkanic traditions (mostly Merino types) became more popular. Herds were owned mostly by the king, aristocrats, cities, and later by the state, but private owners also had and still have their livestock on the steppe. The extensive steppe is parcelled out into separate pastures (ca. 100-800 hectars each) that are used by one herd. Usually 300-800 sheep, and 250-300 cattle form a herd. Animals are kept free without fences, and are herded by a herder with the help of 1-2 dogs.

All interviewed families have a deep rooting in herding: most of their known ancestors were herders. All herders are Hungarians, speak Hungarian, and were born in the region. Most of them follow a more or less traditional way of pasturing. By traditional I refer sensu [8] to social and economic systems that are historically deep, relatively independent of global markets and composed of people whose livelihoods still depend to a substantial degree on the local biomass of the landscape, and who are thus more directly tied to their natural surroundings than city dwellers. Herders spend ca. 200 days per year on the steppe, which has been decreasing sharply in the last decades (usually two herders share the job working in a 24-hours change-over). Herders learnt their herding skills mostly in their own family since their early childhood, and attended school only for 4-6(-10) years. They learnt some modern techniques of agriculture in school, but traditional herding (which was regarded as out-dated) and the botany of the steppes were not taught. The books they have on animal husbandry do not contain local names of plants and habitats, and only describe a few sown grasses and Nitrogen-fixing species and cultivars and the management of artificially created and maintained meadows. I asked several times how often herders had read chapters on pastures and plants in these books, but I could not find a single case. Herders argue that they learnt nothing in school or from books on herding and pasture vegetation except for artificial insemination. Their own folk ecological knowledge reflects this ”ignorance” of modern agricultural and scientific knowledge.

#### Botanical knowledge of herders

**Figure 3:**
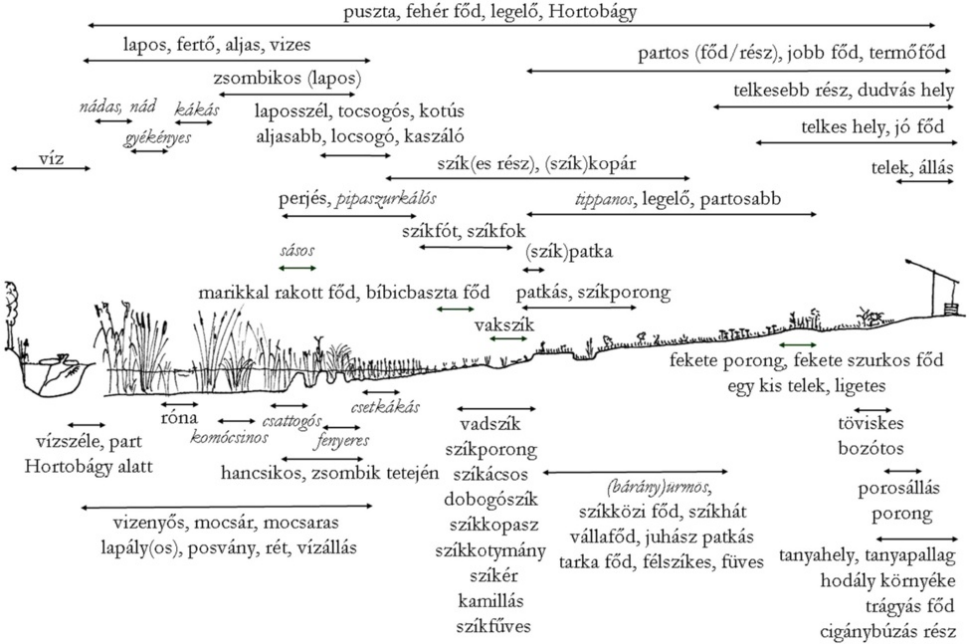
**Habitats along the studied habitat gradient in the Hortobágy salt steppe distinguished by herders.** Arrows indicate degree of inclusiveness of habitat categories. For detailed English equivalents of folk habitat names of this figure see Table 1.

I have studied the knowledge of herders about plants, habitats and the landscape since 2008 with free and semi-structured interviews, free listings, and, as often as possible, through field visits where I participated in herding. I collected 1543 records of habitat names and habitat features, and 1432 records of the knowledge of habitat requirements of wild plant species [28]. Herders distinguish at least 162 folk plant taxa, which is approximately 55% of the ”visible” flora (excluding rare and taxonomically highly similar species) [28]. The habitat classification of herders generated by these non-quantitative methods showed a well developed and somewhat hierarchical structure that was similar (but far from identical) to the botanical classification. Herders distinguished altogether 47-66 habitat categories in the steppe and the surrounding agricultural landscape, of which 37-53 occur on the steppe itself. Many categories overlap or are inclusive with the 7-11 prototypic habitats (Figure 3).

#### Data collection and analysis

For a standardized quantitative analysis of habitat classification, the open picture sort method was used [19]. Of the 37-53 habitat categories herders distinguish in this steppe [29], all the widespread salient habitats were selected for the picture sort, including areas with weed vegetation around sheep/cattle sheds and marsh vegetation, but excluding microhabitats (e.g. ”on the tussock”) and highly inclusive habitat mosaics (e.g. “the whole steppe”). The remaining ca. 20 habitats represent 7-11 main habitat types. More than 200 photographs showing the selected habitat types were taken, from which 23 pictures of different habitat types were shown to 25 herders, 8 botanists, and 8 laymen. Photographed sites were carefully selected to include those habitat features herders regard as salient. In some cases, habitats had to be shown with their neighbourhood habitats (e.g. marsh edge), as this was found to be a salient feature.

Of the 78 previously interviewed herders, the 25 most knowledgeable and communicative ones were chosen for the picture sorts: 23 men, and 2 women; 16 shepherds, 5 cowherds, one wrangler, one swineherd, and two peasants who also kept cattle, horse and sheep but were not professional herders. The mean age was 67,6 (32-85) years. Prior informed consent was obtained before all the interviews, and ethical guidelines suggested by the International Society of Ethnobiology were followed. Picture sorts were usually made by a single herder, but by 2 in two cases, and by 3 persons in two additional cases.

Because the recognition of objects (species, habitats etc.) on pictures is necessarily limited by the nature of the medium, I carefully tested the recognizability of habitats before running the picture sorts. Pictures were of the same size (13*18 cm) and as similar as possible with regard to glossiness cf. [19]. The most representative 53 pictures were shown to 4 herders to select those that they precisely identified with the habitat photographied. I did not use cards with only habitat names on them as suggested by Boster and Johnson [30]. This would have been misleading, as I did not know for certain what herders would have understood from the names on the cards. Most habitats have many synonymous names, while the same name is sometimes offered to different habitats by different people [29].

**Figure 4:**
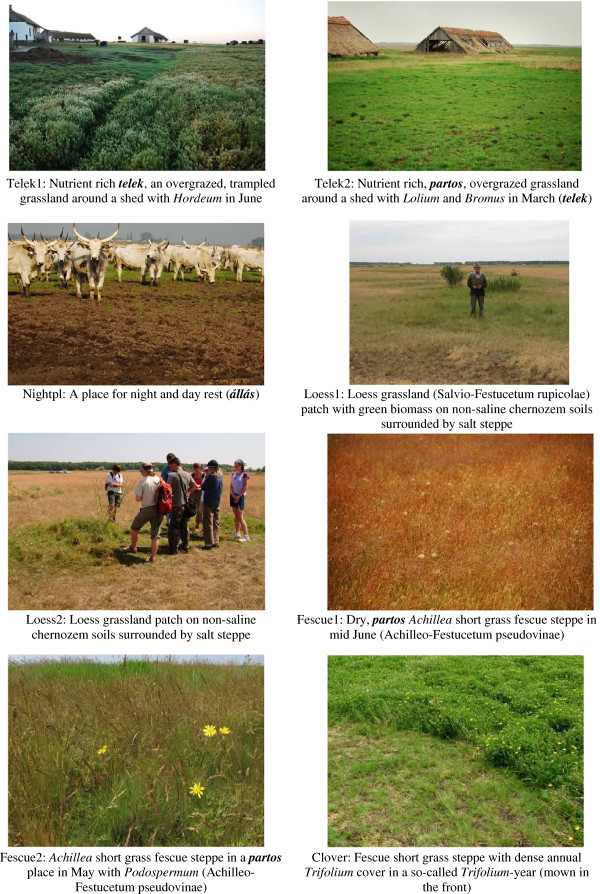
Pictures used in the picture sort exercises: codes and botanical descriptions of dry habitats.

**Figure 5:**
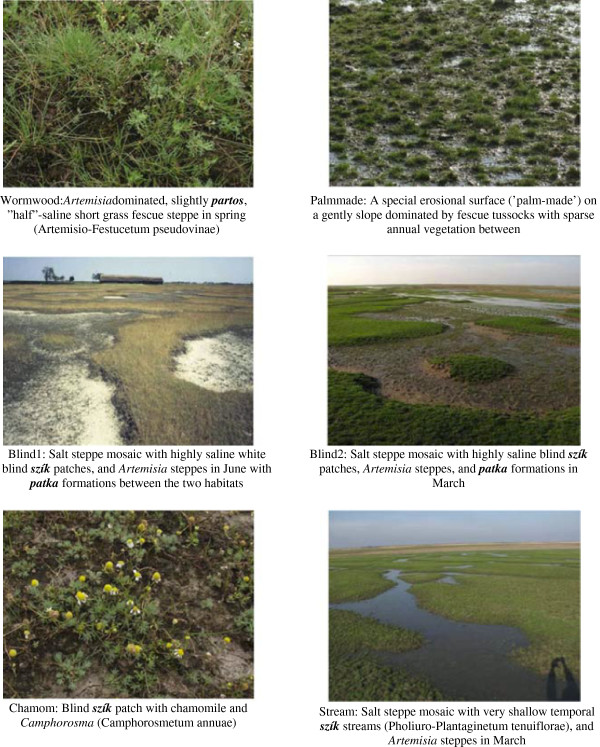
Pictures used in the picture sort exercises: codes and botanical descriptions of salt habitats.

**Figure 6:**
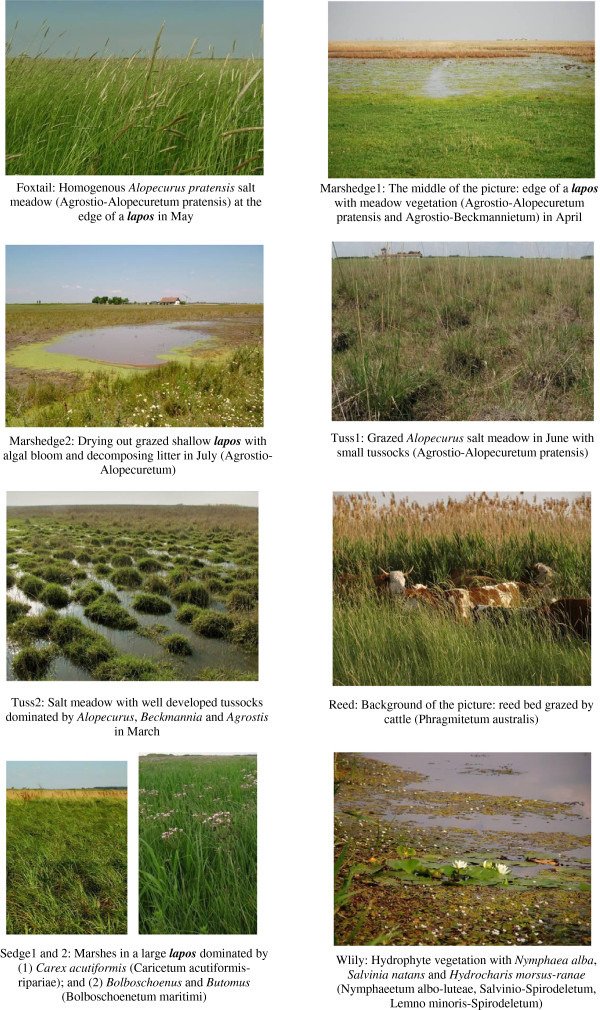
Pictures used in the picture sort exercises: codes and botanical descriptions of wetland habitats.

The final representative set of pictures was selected from the 38 preselected pictures. I chose 2-3 pictures for each main habitat type, as grouping of items might be strongly influenced by how many items are available for any object. Pictures differed in their dominant species, wetness, season, and exact position along the habitat gradient described above. The ’density’ of pictures along the habitat gradient was set as equal as possible (one mistake was made, the picture at the wet end of the gradient (with the water plant *Nymphaea alba*) did not have a ‘pair’). Twenty-three pictures were selected for the picture sort exercise: 8 pictures of habitats with a deep groundwater table, 6 pictures of habitats with highly saline soils, and 9 pictures of meadows and marshes (see Figures 4, 5, 6).

As suggested by Rugg and McGeorge [19], before picture sorts, herders were asked to describe freely the habitats in the pictures in 1-2 sentences. This part of the exercise turned out to be extremely useful, as it gave me the ability to overcome uncertainties or misinterpretations. In some cases, I had to help the herder recognize the scale or the dominant grass species in the picture. Free-ranging descriptions were also useful for further highlighting the attributes herders used to distinguish habitats. In general, working with the pictures in two consecutive rounds also helped prevent hasty decisions. The packet of pictures was shuffled between sorts.

Herders were asked to group pictures that ”go together by habitat, vegetation or grass value”, and not by season or other features of the picture. They were allowed to make as many groups as they wished and include as many pictures in each group using whatever criteria they wanted. After the first sorting, herders were asked if they wanted to change anything, such as move a particular picture from one group to another, create another group, or combine two or more groups, etc. Once the herders were satisfied with their groups, they were asked to justify why they chose to group pictures together.

Data were put into a picture-by-picture co-occurrence matrix. Later, the data from lumper- and splitter-type herders were clustered by selecting those herders who identified the highest or lowest numbers of groups.

The classification by the herders was compared to the classification of the same set of pictures by two contrasting groups of people: botanical experts of the salt steppes (2 women, 6 men, mean age = 37,2 years, 25-65 years) and laymen (5 women, 3 men, mean age = 35,7, 26-45 years). Sample size was limited by the availability of botanists. The laymen, selected were not urban university students, but people living in villages and/or having some experience with Nature (but not with the salt steppe) for example as tourists. To test the representativeness of the limited number of respondents in picture sorts by botanists and laymen, a matrix was prepared from the data of 8 randomly selected herders. This matrix was fairly similar to the one based on the data from all 25 herders. Groupings only slightly differed mostly in the cluster of wet habitats.

The measure of similarity by aggregating sorts depends crucially on the assumption of an underlying cultural consensus [31]. There cannot be fundamentally different bases for classifications among the respondents from any one group, or else we cannot interpret the results. For this reason, cultural consensus was estimated (ANTHROPAC 4.98). All groups (herders, botanists, and laymen) and group combinations were tested. All but two cases exhibited strong fit to the consensus model, supporting the assertion that, despite individual differences, all respondents in the sample belonged to a single culture with respect to this domain (however, less strong fit were found in case of (1) botanists and (2) herders together with laymen, but even in these cases there were no clear evidence for subcultural variation among the groups). I conluded that all groups were sufficiently homogenous for their responses to be statistically relevant.

## Results

### Herder’s classification

Herders usually described pictures in detail (though in some cases they only used one word, the name of the habitat, e.g. ***telek***) (Figures 4, 5, 6 and Table 1). The most important attributes described were soil quality (wetness, salinity), physiognomy and biomass of the vegetation (and their seasonal variation), suitability of the particular habitat for grazing (forage quality of the dominant plant species, seasonality, and seasonal use of the habitat), and present or past land-use. I collected 78 habitat names (including more fixed descriptive phrases) during picture sorts, with 6.5 names per picture on average, and with a minimum of 3 names (for picture 3) and a maximum of 26 names (for picture 11). Since 2008, a total of 181 names for habitats have been collected from herders [29].

**Table 1:** Summary of picture descriptions of herders during picture sort exercises

**Code**	**Herder’s description and name(s)**
telek1	rich, fertilized soil (manured by resting animals), fat and black earth with *Hordeum hystrix* (***telek*** – rich area around farmstead, ***tanyakörnyék*** – area around the shed/farmstead*,****telekszél*** – edge of ***telek***, ***állás****–* resting place for the animals*,****tanyaudvar, tanyapallag****–* ground around the farmstead, ***dudvás rész*** – manured place, ***cigánybúzás rész*** – area with *Hordeum*)
telek2	good rich soil, strong earth with mixed grass, the most ***partos*** (lit. on the shore), all animals stop here for grazing, but fescue dies out, after driving out in spring, first we grazed the litter, than some places were left for mowing, it has a good mixed hay, in summer this area is hot, grass burns, thorny plants abound, e.g. *Xanthium spinosum* (***telek***, ***állás környéke*** - around the ***állás***, ***tanyapallag***, ***tanyakert környéke***, ***partos hely*** – ***partos*** place, ***telekszéle***)
nightpl	good quality earth, like on the ***telek*** (***állás***, ***gulyaállás*** – resting place for cattle, ***telkes főd*** – with earth like on the ***telek***)
loess1	richer black earth, ***partos***-like, pasture is better in patches, soil keeps water longer, grass is mixed, weedy, ancient pasture, not saline, *Urtica*, *Elymus*, bushes and mushrooms, this area got manure or it is an abandoned ***állás*** or was ploughed in the past, others: it is natural, not made by mans hand (***fekete porong*** – black elevated area, ***partos***, ***telkesebb rész***, ***legelő*** - pasture, ***ligetes terület*** – patchy area, often no habitat name was mentioned)
loess2	better, richer earth, plants want to live longer, the ’lifting power’ of the soil is stronger, grass is more mixed, ***partos***, like ***telek***, there is no salt, *Carduus*, *Cardaria*, *Euphorbia*, *Urtica*, *Trifolium*, *Eryngium*, and mushrooms, a farm or well was here, or manure was deposited, or it was disturbed, others: Nature created it (***fekete porong***, ***hátas főd*** – earth like a back, ***nagyon kicsi telek*** – very small ***telek***, ***ligetes fót*** – patchy, ***legelő***)
fescue1	this is all fescue, ancient saline pasture, but with a bit ***telek***-like soil, a bit ***partos***, the strongest, most useful grass, tiny hay (means: very good, not like the long *Alopecurus*), cattle is best if eating this, here on the picture sun burnt it (***tippan*** - fescue, ***tippanos*** – area with fescue, ***rendes legelő*** – good pasture, ***szík*** - salt)
fescue2	better, a bit ***telek***-like earth (better than the one with *Artemisia*), not as saline, on the back, ***partos***, mixed, denser, sometimes mown, valuable grass mixed with less valuable, fescue, *Podospermum*, *Trifolium* (usually no names, ***fekete porong***, ***legelő***)
clover	richer, good pasture on ***partos*** place or at the edge of a marsh, but not in each year, somewhat better saline soil (not white), mixed valuable grass, but dangerous (bloats up and kills the sheep) (***bodorkás rész*** – area with *Trifolium*, ***hátas rész***, ***laposszél*** – edge of the ***lapos***, legelő, ***kaszáló*** – area for hay)
wormwood	this is the typical fescue pasture of the Hortobágy, salty, but a bit ***partos***, not the true ***szík***, not blind ***szík***, but not really on the black earth, gray earth, a bit wet, not very valuable, soil structure is not as good, semi-grassy salty place, fescue grows in tussocks (often does not get a name, ***bárányürmös, ürmös rész*** – with *Artemisia*, ***tippanos***)
palmmade	very saline earth, can not produce more, lapwings like it, and also skylarks, fescue grows in small tussocks, only fescue, nothing grows with it, wetter and better in spring, but does not last long, in drought it is barren, we do not like it (***marikkal rakott főd*** – ‘palm-made’ earth, ***bíbicbaszta főd*** – earth f…cked by the lapwing, ***bíbickocogtató*** – lapwing run, ***hancsik/zsombikos*** - tussocky)
blind1	typical saline pasture, but only half pasture (you can only graze half of the area), good for nothing earth, no calory, water-logged, salt depletes it, mud stucks between the nails of sheep, wind blows the white dust, animals lick the salt, sheep does not like to graze here, only the strong fescue grasses on the ***partos*** place (names of the whole mosaic: ***szík*** – salt (salty place), ***szíkfoltos, szíkfokos terület*** – area with salty patches, ***tarka főd*** – patterned earth, ***szíkpatkás legelő*** – pasture with ***patka***; habitats in the depressions: ***szík***, ***szíkfót*** – ***szík*** patch, ***szíkes főd*** – salty earth, ***szíksó*** - salt, ***fehér szík*** – white ***szík***, ***szíksavas főd*** – earth with salt, ***szíkfok***, ***szíkkotymány*** – ***szík*** brew; ***partos*** habitats: ***füves*** - grassy , ***tippanos***, ***szíkporong*** – elevated ***szík***, ***szíkközi főd*** – earth between ***szík***, ***szíkpadka*** – (the special geomorphological feature), ***szíkhát*** – ***szík*** back, ***legelő***, ***termőfőd*** – earth that bears, ***partos***, ***szíkpart***, ***gyep*** - grassland)
blind2	earth with a bad structure, highly salty, bad even after the withdrawal of water, not good as a pasture, disgraceful, if wet, in depressions: small grasses grow, salt depletes, barren, water-logged, on ***partos***: these are grasslands with fescue and *Artemisia*, green pasture that is eroded (the mosaic: ***szíkes*** - salty, ***szíkpadkás rész***, ***szíkfokos***; depressions: ***vakszík***, ***vadszík*** – wild ***szík***, ***szík***, ***szíkfót***, ***szíkporong*** –salty depression; on ***partos: tippanos rész***, ***porong***, ***gyep***, ***partos***)
chamom	true salty area with chamomile, and with this tiny red creeping plant (*Camphorosma*), not blind szík, a bit wetter szík, as a pasture nothing!, unimprovable, strong soil, poor earth without neat grass (***kamilla*** - chamomile, ***kamillás/szíkfűves*** – with chamomile, ***szíkfót***, ***szíkes rész***, ***szíkfok***, ***szíkes talaj***- salty soil)
stream	water-worn, eroded, water is coming (fleeing!) from the ***partos*** for centuries, after snow melting and rain water stays, but in summer after two days tiny green grass grows, animals like it, otherwise a bad pasture, salty, sinking (***szík***, ***ér*** – stream, ***kis erecskék*** – tiny streams, ***szíkes erek*** – salty streams, ***vízér*** – water stream, ***szíkfok***, ***szíkpatkák***)
foxtail	true wet grass, good pasture, others say: bad pasture, worth nothing, grass withers quickly, wet area, not ***partos***, after rains it is water-logged, but later dries out (***perje*** - *Alopecurus*, ***perjés/pipaszúrkálós*** - with *Alopecurus*, ***lapossas rész*** – a bit like ***lapos***, ***aljas rész*** – depressed place, ***laposszél*** – edge of ***lapos***, ***kaszáló*** – area for hay)
marshedge1	water starts here, water runs here, shallow water, with *Alopecurus*, you can make hay, if not grazed, quickly warms up, horses like it, they hide here from horseflies, it can turn wild if not grazed (***laposszél***, ***mocsaras rész*** – marshy area, ***vízállás***, ***tocsogós*** – (with shallow water), ***mocsár*** - marsh, ***kotyványos rész*** – mud brew, ***lapos***, ***ér***)
marshedge2	water-logged for long, in warm water grass rots, and it has a bad taste, worthless, smelly, with algae and *Lemna* (***lapos***, ***fenék*** - bottom, ***vizes rész*** –place with water, ***pocsmány*** (see ***kotyvány***), ***locsogó*** – (with shallow water), ***laposszél***)
tuss1	area at the edge of ***lapos***, poor hay, with *Alopecurus*, water-logged for longer, but dries out in summer, not very salty, but not rich, either, tussocky, an earthworm creates it, animals trample, horses like to graze here, but sheep do not like (***zsombikos lapos*** – ***lapos*** with tussocks, ***laposszél***, ***pipaszúrkálós***, ***vízállás*** –water stands)
tuss2	tussocky, the earthworm builds it, useless pasture, worth nothing, water-logged pasture, ducks hide, lapwings breed on the tussocks, in poor dry summer animals like to graze here, if there is nothing on the ***partos*** (***zsombikos***, ***zsombikos lapos***, ***lapos***)
reed	wet, muddy, dense area with reed, poor pasture, cattle eat some of it, they hide here from horsefly (***nádas*** – with reed, ***lapos***)
sedge1	wet, marshy area, worthless as a pasture, *Typha*, *Bolboschoenus*, *Schoenoplectus*, *Butomus*, *Iris* (***lapos***, ***laposszél***, ***kákás*** – with *Schoenoplectus*, ***lapossas rész***, ***vizenyős hely*** – place with water, ***mocsaras rész***)
sedge2	very ***lapos***, wet area, water-logged throughout the year, worthless, good for nothing, useless plants, only to fill the belly, if nothing is left on the steppe, animals ate it, we stood in front of them, they ate up everything, *Carex*, *Bolboschoenus*, *Typha*, *Schoenoplectus*, *Rumex* (***sásos*** – with *Carex*, ***lapos***, ***vizes terület***, ***gyíkínyes*** – with *Typha*, ***laposszél***, ***csetkákás*** – with *Schoenoplectus*)
wlily	deeper water but flows, marshy, does not dry out, it is not a pasture, animals do not come here, *Nymphaea*, *Nuphar*, *Lemna*, *Ceratophyllum*-like plants float on the water, dangerous (***víz*** - water, ***nyílt víz*** – open water, ***mocsaras rész***, ***vízi növényzet*** – water plants, ***nagy lapos*** – big ***lapos***)

Text was mostly translated literally. Habitat names and their equivalents are given in parentheses when first mentioned.

**Figure 7:**
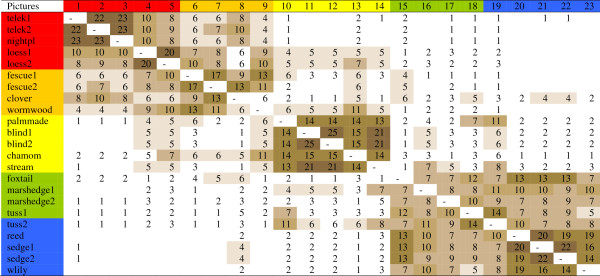
**Matrix of open picture sorts of Hortobágy salt steppe habitats (23 pictures, 25 herders).** Numbers indicate how often pairs of pictures were put into the same group of pictures. Colours indicate different habitats (red: non-saline habitats on black earth at the higher end of the habitat gradient, orange: fescue dominated short grass steppes, yellow: highly saline habitats usually with sparse vegetation cover, green: tall-grass salt meadows with seasonal water-cover, blue: water-logged marshes with reed, sedge etc.).

The picture-by-picture matrix of the 25 herders is shown in Figure 7, for the names of groups see Table 1. Three large groups were identified by the herders: (a) more fertile habitats at the higher parts of the habitat gradient of the steppe (***partos***, lit. on the shore) (pictures 1-9); (b) saline habitats in the middle of the habitat gradient (pictures 10-14) (***szík***, lit. salt or saline place), and (c) meadows and marshes in the lower parts of the habitat gradient (pictures 15-23) (***lapos***, lit. flooded). Saline habitats were fairly isolated from the rest. Habitats with the most fertile soils (1-3) were only exceptionally grouped by some people with saline or wet habitats. Marshes (19-23) were usually kept together with meadows, and were rarely grouped together with saline habitats, and almost never with fertile ones. Loess grasslands (4-5) formed a separate subgroup of habitats with low groundwater table. The short-grass fescue steppes (with *Artemisia* or *Achillea*, 6-9) did not form a well separated group. Saline *Artemisia* steppes (9) were more often grouped with non-saline *Achillea*-type fescue steppes (6-8) than with highly saline habitats (10-14). The habitat dominated by annual *Trifolium* species (8) was partly grouped with meadows or marshes, while the so-called ‘palm-made’ fescue grassland (10) to the tussocky meadows (18-19). Drier *Alopecurus* meadows (15) were more often grouped with marshes (18-22) than with fescue grasslands, and even less often with highly saline habitats or the more fertile ones. Meadows (15-18) were not more often grouped together than with marshes. The deepest marsh habitat (with *Nymphaea alba*, 23) often formed a group by itself. In summary, herders identified three large and 5(+2) small groups. For group justifications see Table 2.

**Figure 8:**
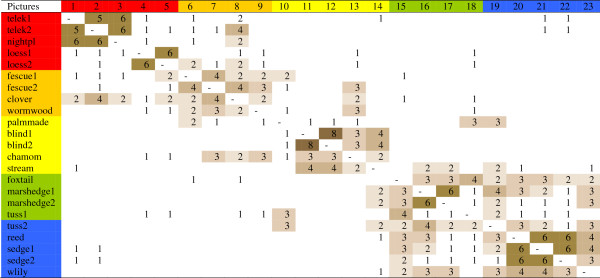
Matrix of open picture sorts of the 8 splitter-type herders.

**Figure 9:**
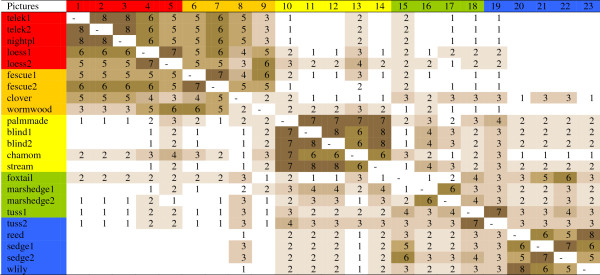
Matrix of open picture sorts of the 8 lumper-type herders.

Splitter-type herders identified 6(+1) groups (Figure 8). The most fertile habitats around animal sheds (1-3) were separated sharply from loess grasslands (4-5). Short-grass fescue steppes (6-8) formed the next group. Saline habitats were isolated from meadows, and marshes formed the last group. Lumper-type herders classified habitats into three large groups (Figure 9) divided into 7(+1) small ones, as tussocky habitats (18-19) were separated from other meadows (15-17). The gap between saline (10-14) and more fertile habitats (1-9) was bigger than the gap between saline and wetland habitats (15-23).

### Botanists’ classification

**Figure 10:**
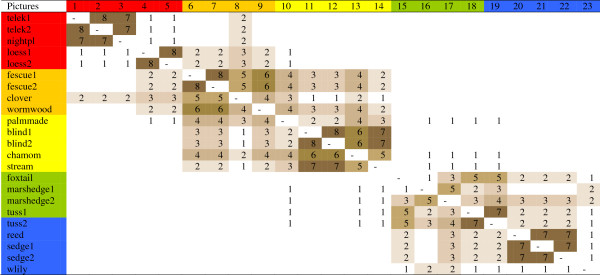
Results of open picture sorts of salt steppe habitats by 8 botanists (experts of saline steppes).

Botanists identified 6 well-defined groups (Figure 10): fertile, weedy, degraded grasslands (1-3), loess grasslands (4-5), short-grass fescue steppes (6-9), highly saline habitats (10-14), meadows (15-19), and marshes (20-23). Gaps were usually conspicuous between the groups. Three of the groups had a more complex structure: (a) the *Achillea* and *Artemisia* dominated short-grass fescue grasslands showed some separation; (b) the group of highly saline habitats was heterogenous and was much more connected to fescue steppes than to meadows; (c) meadows formed a loose group partly connected to marshes. All wet habitats together formed a large group, as did all saline and fescue-dominated habitats. Justifications often contained names of vegetation types, or descriptive phrases with information on the vegetation itself.

**Table 2:** Herder’s justifications of the groups of pictures (typical examples)

**No.**	**Herder’s description of the cluster**	**1**	**2**	**3**	**4**	**5**	**6**	**7**	**8**	**9**	**10**	**11**	**12**	**13**	**14**	**15**	**16**	**17**	**18**	**19**	**20**	**21**	**22**	**23**
1	***állás*** places, these form a separate group!	X	X	X																				
2	not wet, not salty, true ***telek***, first class	X	X	X		X																		
3	***telek***, ***állás*** or edge of it, good earth	X	X	X					X															
4	this is good, good soil structure, better than the salty	X	X	X			X	X	X															
5	better, ***telek***-like areas	X	X	X	X			X	X															
6	good grass		X		X	X			X															
7	these are all usefull grasses!	X	X		X	X	X	X	X	X														
8	mixed grass, better quality, no blind ***szík***				X	X	X	X		X														
9	like ***telek***, better, fatter pastures				X	X		X	X															
10	with fescue on the ***partos***, all with fescue						X	X	X	X														
11	this is ***szík***, with different grasses, a perfect group!						X	X		X														
12	true pasture, better or worse, but animals can graze	X	X	X	X	X	X	X	X							X	X	X	X	X				
13	with fescue						X	X		X					X									
14	not a salt patch: fescue and *Alopecurus*						X	X								X								
15	not wild ***szík***, second class white earth						X	X	X	X				X										
16	spring pasture: *Trifolium* and chamomile							X	X	X				X										
17	these are pastures with grasses							X	X							X								
18	*Alopecurus* for hay, *Trifolium*, but good also for grazing							X	X							X								
19	***szík patka*** and ***szík*** streams				X						X	X	X											
20	salty with fescue, these plants all need the same habitat, all are salty places						X			X	X	X	X	X	X									
21	***szík*** patches, all like salty earth or the edge of it									X	X	X	X	X										
22	true blind ***szík***, salty										X	X	X	X	X									
23	with ***szíkfok***, tussocky, salty										X	X	X	X	X			X						
24	salty places				X	X					X	X	X	X	X					X		X		
25	not ***lapos***, not ***partos***, you can not drain												X	X	X				X	X				
26	with *Alopecurus*, does not fit in any groups, though wet															X								
27	wet areas, not ***partos***								X		X	X	X		X		X	X	X	X	X	X	X	X
28	with *Alopecurus*															X	X							
29	tussocky, and wet														X		X	X						
30	water-logged pasture, dry in summer														X					X	X	X		
31	water plants, they go into one group!															X					X	X	X	X
32	tussocky, this is ***lapos*** with reed															X		X			X	X	X	X
33	bad grass, *Typha*, worthless															X			X	X	X	X	X	
34	water-logged places, marshy areas																	X	X	X	X	X	X	X
35	totally wet, never dries out																		X	X	X			X
36	all is ***lapos***, water does not go out, only in strong drought																			X	X	X	X	X
37	it does not have a pair, deep water																							X

### Laymen’s classification

**Figure 11:**
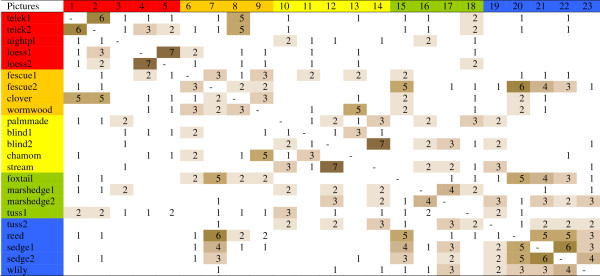
Results of open picture sorts of habitats by 8 laymen who did not have knowledge on saline steppes.

Laymen grouped habitats in a less coherent way (Figure 11). Though values along the diagonal of the matrix tended to be higher, than further from the diagonal, groups were not well structured. The most developed group was formed by marsh habitats. Short-grass fescue steppes (6-9) formed a loose group. Often only pairs of pictures formed the ”group” (e.g. 1-2, 4-5, 6-7, 8-9, 11-12, 16-17, 20-22). In some cases, habitats lying far from each other along the gradient formed groups (e.g. 7-(20-22)-15; (1-2)-8). In general, three main salient features were mentioned in justifications: dryness, greenness, and wetness.

## Discussion

### Herders’ classification of salt steppe habitats

All herders could attach a habitat name to any pictures. Habitats were grouped into three higher-order categories: (1) high forage quality pastures on the higher parts of the habitat gradient, (2) low forage quality pastures in the middle on saline soils, and (3) meadows and marshes with low quality grass. Hierarchy below this level was much less developed. It was unexpected that the most common and most important pastures (the short grass fescue steppes) did not form an isolated cluster (though herders say: ”Fescue is the first grass on the steppe, almost as good as alfalfa!”). Fescue steppes were kept together with the even more productive but not as widespread loess steppes, and rarely with drier *Alopecurus* meadows. These habitats were the most important pastures for the herders. Patches with annual *Trifolium* species had a tendency to be grouped with *Alopecurus* meadows, as these important species also occur there. The close connection of loess steppes with the most saline habitats seems to be an artifact caused by the surrounding saline grasslands. My previous experiences suggested that loess grasslands and short-grass fescue steppes are well separated by herders (see Figure 2), but herders recognized loess steppes on pictures only if shown with their contrasting surrounding landscape. However, it is also possible, that loess steppes were grouped together with fescue steppes because they belong to the short-grass pasture mosaic (habitats on pictures 1-14). The ‘palm-made’ fescue habitat was often grouped with tussock meadows based on physiognomic similarity – both being somehow tussocky (though tussocks in the meadows are not 5-10, but 30-60 cm tall).

As I expected, sharpness of delimitation of habitats changed within the gradient (Figure 7). The most well defined group was formed by the saline habitats. Habitats were ordered in a shallow hierarchy that seemed to be shared among herders. The herders’ comments during picture sorts (e.g. ”Where should I start?”; ”Does this fit in this group?”) suggest that this hierarchy is not necessarily conscious. Sorting most often started with marshes or highly saline habitats, and was finished with the less salient short-grass fescue steppes. In most cases, justification of groupings was not difficult for them.

Shepard et al. [6] found that abiotic and biotic factors of named habitat categories are considered somewhat independently, thus the habitat classification is not organized into a single, unified hierarchy. Fleck and Harder [12] also documented two more or less independent classifications (one based on geomorphology, and one on vegetation), as did Meilleur [15] in the French Alps, though there the relations among the different criteria were unclear. In our steppe landscape abiotic and biotic classifications seemed to be much less independent, consequently habitats were ordered into a single classification system.

### Comparison of habitat classifications performed by herders, botanists and laymen

As opposed to my expectations, botanical classification was not more structured than that by herders, though the large clusters were more isolated in the botanical sorting. Both groups sorted habitats according to their vegetation structure, salinity, water cover and nutrient richness. Dominant species seemed less important.

Herders grouped short-grass *Achillea*–type fescue steppes together with the more fertile habitats (loess steppes and ***telek***), while botanists grouped them with the saline ones. There is a long-standing debate in Hungarian phytosociology, whether short-grass *Achillea*–type fescue steppes belong to saline habitats or to loess steppes cf. [23,32]. Species composition of *Achillea*–type fescue steppes is more similar to degraded loess steppes, while deeper soil layers are saline. This debate was reflected in the contrast between herder and botanist appreciations.

Loess grasslands and degraded, nutrient rich grasslands around sheds were more clearly separated by botanists, as nutrient rich stands are dominated by weeds, while loess grasslands often harbour rare steppe specialists (e.g. *Phlomis tuberosa, Thalictrum minus*), which thus give them more distinctiveness to botanists.

Classification of splitter-type herders resembled botanical classifications, though herders separated the fescue dominated habitats from highly saline habitats more often. It seems that the goal for the botanists was to maximize inductive potential for botanical features (e.g. occurrence of rare loess or salt steppe species) and to categorize nature, regardless of human (land)use interest (e.g. pasture value, general agricultural productivity) cf. [33].

As expected, laymen’s classifications were considerably different from the other two. Justifications showed that laymen grouped habitats according to wetness, greenness/suitability for grazing, and dryness. As Boster and Johnson [30] experienced in sorts with fishermen and laymen, laymen tended to focus on morphological features of fish species visible in the pictures, since they sorted species with which they had limited knowledge. I also found that pictures with similar views were often grouped together (correctly e.g. 4-5, 10-12, or mistakenly e.g. 7 and 15). Some important habitat features were not at all visible on pictures (moderate salinity of soil, factors behind grass density – wetness or nutrient richness, and often the neighbouring habitats). The large group of saline habitats prominent in the classifications of herders and botanists is missing from laymen’s classifications, since ‘salinity’ of soil was only recognizable for laymen on pictures 11-13 (vegetation with chamomile). Much less aggregation of values along the diagonal also indicates that laymen did not recognize the habitat gradient of the salt steppe.

I expected and found high correspondence among the classifications by herders, botanists and laymen. Herders, botanists and to a lesser extent laymen tended to recognize similar units and larger groups along the habitat gradient. In all classifications, there were clusters of wetlands, ”good grass” habitats and dry or saline habitats. Results of the cultural consensus analysis also showed that herders, botanists and laymen all belonged to the same culture with respect to this domain. According to Berlin [1], and Bailenson et al. [34], several factors can underlie similar classifications: (1) salient features correlate; and/or (2) the same salient features are noted and used as a basis for sorting. In my case, both explanations were feasible: (1) salinity may correlate with the density of grasslands, height of vegetation; wetness with vegetation height; greenness with wetness and nutrient richness; and (2) justifications showed that all three groups of people regarded wetness, abiotic stress as expressed in grass height, grass density, and greenness as the most salient features. Herders used these features to assess grassland quality (”How good this pasture would be for my animals”), botanists induced the occurrence of other plant species, while laymen did not have a goal other than to group the pictures as best they could in accordance with the instructions received. Herders and botanists might have also been able to predict similar habitat features invisible on the pictures (soil type, coexisting plant species, precipitation events prior to the date the photos were taken, etc.), which made their sortings more similar.

### Specificities of folk habitat classifications

Habitat patches in a landscape are perceived by humans by walking/working in it (woodlands), or walking/working on it (grasslands). From look-out points (or on remotely sensed images), hundreds or thousands of habitat patches can be perceived. However, habitat patches are less discrete elements of nature than species populations, as habitats belong to different continua [17,35]. In case of habitats only variants exist in nature, that are arranged around prototypes [36], sensu [37]. Continua are key determinants also of folk landscape classifications [7,8,20].

We might ask, how many categories are optimal for a landscape ethnoecological classification? Humans usually ”map” the landscapes at a 1: 10 000-25 000 scale, which is mainly determined by the size of humans and the size of the land they know personally. This scale is one of the determinants of distinguished habitat types. Hunn and Meilleur [7] suggested that distinguishing 25 habitats is the ”average” for a traditional community/landscape. Although the potential number of habitat types is infinite, the number of habitat categories is highly dependent on the depth and extent of the study. In Gyimes (Eastern Carpathians, Romania), for example, Babai and Molnár [38] found at least 142 folk habitat categories that exist in a single valley (ca. 60 km^2^). This high number (higher, than ever found elsewhere, cf. [7]) results not only from the property of the landscape (highly diverse mountainous area), nor the deep ecological knowledge of the local Csángó people, but it is also the result of the depths of the investigation. Moreover, there were usually several more-or-less synonymous names for one habitat category. People distinguished habitats at different spatial scales, which also caused overlaps. These factors prevented the determination of the exact number of folk habitat types. I question, therefore, whether it is possible at all to determine the exact number of distinguished folk habitat categories in a region. This is the reason I previously wrote that 37-53 habitat types are distinguished in the Hortobágy steppe landscape.

Hunn and Meilleur [7] regard habitat categories as “natural” in the sense that particular species of plants and/or animals are predictably associated with certain habitat patches. Phytosociologists and vegetation scientists also argue that basic vegetation units exist in nature as real entities and do not represent merely abstract categories [17,18]. However, phytosociological and ethnoecological evidence suggest that basic cuts in categorizations are made frequently at subjectively weighted discontinuities. Consequently habitat delimitations along continua are often at least somewhat subjective and arbitrary [29,38]. It follows that several, more or less different and natural classifications can be developed for the same landscape depending on the motives, the backgrounds, and the experiences of the classifiers. In my matrix of picture sorts, values farther from the diagonal (and justifications, too, see Table 2) suggest that even within the herders somewhat different alternative classifications existed. I argue that a single best habitat classification of an area does not exist (cf. classification-based categories, [20]), but I would argue, there will always be a core of agreement.

Even if the rules used to create a categorization are fixed, the selection of prototypic types sensu [37] will always be somewhat subjective [1,35,39,40] and will depend on how many landscapes and how deeply the person doing the categorization knows. It will also depend on how many and what type of spatial and temporal transitions exist in the landscape [39]. In mapping exercises with botanists, we found that delimitation and characterization of patches differ among mappers even if habitat classification, scale and mapping algorithms are fixed [39,41].

Studying folk habitat classification seems to be more difficult than studying species classification. Habitat knowledge is more implicit and more variable, and thus more difficult to elicit [9,20,21]. During discussions with traditional knowledge holders, one has to check what a person means by a habitat name. Dozens of field reconnaissances are necessary to reconstruct the variations and semantic differences in meanings cf. [9,13,42]. For a detailed study of folk habitat knowledge, the researcher also has to have a deep and intimate knowledge of the landscape. Without it, conversations will be superficial cf. [43]. A cross-cultural comparison of folk habitat classification is limited by the lack of a comprehensive, intercontinental habitat taxonomy [20], whereas the low proportion of binomials, and the many synonymous names limit purely linguistic approaches cf. [44].

Despite all these difficulties, conducting landscape ethnoecological research will inevitably result in a deeper understanding of human cognition at a supraspecific level of biological organization, where natural discontinuities are less sharp than at the level of scientific species [7,11,45,46]. Additionally, folk habitat knowledge contains a wealth of information (and wisdom) about the landscape, which can be used in vegetation science, nature conservation and environmental education [47]. Herders in Central Europe are walking encyclopaedias of landscape knowledge sensu [43], and most of the pages of these are as yet unread by biologists and nature conservationists. Fortunately, at least in the Hungarian steppe, habitat related traditional ecological knowledge is fading at a lower rate than other traditional knowledge since holders of this knowledge still need it for their everyday life.

## Competing interests

The author declares that he has no competing interests.

## Authors’ contribution

This is a sole authored paper.
